# A Novel Homozygous *MC2R* Variant Leading to Type-1 Familial Glucocorticoid Deficiency

**DOI:** 10.1210/jendso/bvac058

**Published:** 2022-04-08

**Authors:** Idris Mohammed, Basma Haris, Khalid Hussain

**Affiliations:** 1 Division of Endocrinology, Department of Pediatrics, Sidra Medicine, Doha, Qatar; 2 College of Health & Life Sciences, Hamad Bin Khalifa University, Qatar Foundation, Doha, Qatar

**Keywords:** MC2R, ACTH, adrenal insufficiency, familial glucocorticoid deficiency, next-generation sequencing

## Abstract

**Context:**

Type 1 familial glucocorticoid deficiency (FGD) (OMIM #607397) is a rare autosomal recessive disorder due to mutations in melanocortin-2-receptor (MC2R) gene encoding the G protein-coupled adrenocorticotropic (ACTH) transmembrane receptor.

**Objective:**

The aim of the study is to describe 2 siblings born to a healthy consanguineous family presenting with clinical and biochemical features of FGD, harboring a novel homozygous MC2R variant.

**Methods:**

Both patients are siblings born at term via normal delivery with normal birth weights. The first sibling presented with symptoms of hypoglycemia, repeated episodes of infections starting from 2 days of age. At 18 months of age, low serum cortisol was found, and he was started on hydrocortisone replacement therapy. The second sibling developed hypoglycemia on day 1 after birth, investigations revealed low serum sodium and cortisol levels and was also commenced on hydrocortisone treatment. Whole exome sequencing (WES) and in vitro functional studies on cell line transfected with wild-type and mutant plasmid clones were undertaken.

**Results:**

WES revealed a novel homozygous missense mutation c.326T>A, p.Leu109Gln in the MC2R gene. In-silico prediction tools predicted the effect of this mutation to be deleterious. In vitro study using HEK293 cells transfected with MC2R wild-type and mutant clones showed a defect in protein expression and cAMP generation when stimulated with ACTH.

**Conclusion:**

Homozygous semiconserved p.Leu109Gln mutation disrupts cAMP production and MC2R protein expression leading to ACTH resistance. This study provides additional evidence that this novel pathogenic variant in MC2R results in FGD phenotypes.

Familial glucocorticoid deficiency (FGD) (OMIM #202200), also known as isolated glucocorticoid deficiency, or hereditary unresponsiveness to adrenocorticotropin hormone (ACTH), is an autosomal recessive disorder due to a failure of the action of ACTH to stimulate the adrenal gland to produce glucocorticoids [1]. FGD was first reported in 2 sisters in 1959 by Shepard and colleagues [2] and is characterized by high plasma ACTH levels and severe cortisol deficiency. If left untreated, FGD can be lethal and may lead to death due to hypoglycemia, increased susceptibility to infections, hyperpigmentation, and seizures [3].

FGD is a heterogeneous disorder with multiple subtypes. The 2 most common subtypes are FGD type 1 (OMIM #607397) and FGD type 2 (OMIM #609196). Mutations in melanocortin-2 receptor (*MC2R*) cause FGD type 1 while mutations in its accessory protein, melanocortin-2 receptor accessory protein (*MRAP*) lead to FGD type 2. Mutations in both *MC2R* and *MRAP* genes account for 40% to 55% of FGD cases [4]. Even though MC2R and MRAP are the 2 most common causes of FGD, due to advances in genome sequencing, recently several other genes which lead to adrenal insufficiency have been identified. These genes include minichromosomal maintenance-4 deficiency (*MCM4*) [5], nicotinamide nucleotide transhydrogenase (*NNT*) [6], steroid acute regulatory (*STAR*) [7], thioredoxin reductase 2 (*TXNRD2*) [8], and sphingosine-1-phosphate lyase (*SGPL1*) [9].

Five melanocortin receptors (MC1R to MC5R) have been identified so far. These receptors consist of 7 transmembrane G protein coupled receptors (GPCRs), which are closely related in structure and share homology at the amino acid level [10]. Melanocortin receptors show overlapping specificities to ligand affinity and they bind to several melanocortins, α-MSH, β-MSH, γ-MSH, δ-MSH, and ACTH; only MC2R shows high selectivity for adrenocorticotropic hormone (ACTH) [11]. The human MC2R, also known as ACTH receptor, located on chromosome 18p11.21, is the smallest GPCR, encoding a 297-amino acid protein with a molecular weight of around 33 kDa [12]. MC2R is predominantly expressed in all 3 layers of the adrenal cortex; zona reticularis and zona fasciculata stimulate the secretion of glucocorticoids while zona glomerulosa stimulates the secretion of aldosterone. MC2R activation by ACTH leads to an increase in cyclic adenosine monophosphate (cAMP) and protein kinase A, subsequently activating the steroidogenic enzymes expression pathway [13]. MC2R inactivating mutations lead to low levels of cortisol and high levels of plasma ACTH, consequently manifesting features of FGD [14]. There is only 1 MC2R activating mutation (F278C) reported in literature which is associated with Cushing syndrome [15]. Here we report a novel homozygous loss-of-function MC2R mutation in 2 siblings with hypoglycemia, recurrent infections, and low serum cortisol level.

## Case Description

### Patient 1

Sibling 1 is a 4-year-old boy who was born at term via normal delivery with a birth weight of 3.45 kg. The parents are first-degree cousins. The mother was well during the pregnancy. The patient was admitted to the neonatal unit at for hypoglycemia at a hospital outside Qatar and was evaluated but no cause was found as per the parents. He developed repeated episodes of neonatal sepsis starting at the age of 2 days with recurrent episodes of hypoglycemia and infections. At 18 months of age, the patient became unwell and was admitted in our center with hypoglycemia. Investigations confirmed a high plasma ACTH and low cortisol level. Interestingly the serum thyroxine level was also low and the thyrotropin (thyroid-stimulating hormone; TSH) was found to be high. The patient was therefore started on hydrocortisone replacement therapy (12 mg/m^2^/day) and thyroxine supplementation (25 mcg daily). The serum insulin and C-peptide levels were undetectable. There was no failure to thrive or any dysmorphic features, but the patient had increased pigmentation which improved after starting replacement therapy with hydrocortisone.

### Patient 2

Sibling 2 is the second child of the same family. She was born at 38 weeks of gestation via normal vaginal delivery with a birth weight of 3.54 kg. The mother had gestational diabetes in this pregnancy that was managed with dietary control only. The patient developed hypoglycemia (2.1 mmol/L) on day 1 after birth. She was monitored closely, and investigations revealed low serum cortisol levels as well as high plasma ACTH levels. A short Synacthen test showed no response to cortisol, so the baby was started on hydrocortisone treatment (12 mg/m^2^/day). Her serum TSH and plasma aldosterone levels were elevated.

Table 1 summarizes the biochemical investigations in both siblings.

**Table 1.** Clinical investigations of the 2 patients

Test	Value		Reference
	Sibling 1	Sibling 2	
Age at time of test	18 months	4 days	
Plasma ACTH	>1500	952	5-60 pg/mL
Serum cortisol	<22	<22	69-632 nmol/L
Plasma aldosterone	151	2800	194-2579 pmol/L
Serum sodium	137	136	135-145 mmol/L
Serum potassium	3.7	5.2	3.5-5.2 mmol/L
Serum bicarbonate	23	21	21-28 mmol/L
Serum chloride	104	99	95-110 mmol/L
Serum TSH	9.23	10.7	0.4-4 mIU/L
Serum free T4	9.1	17.6	9.5-17.8 pmol/L
Plasma renin activity (PRA)	3.7	Not done	1.5-3.5 ng/mL/hr

## Methodology

### Whole Exome Sequencing

After obtaining written consent from the family, blood was collected from the patients and their parents for whole exome sequencing (WES). Genomic DNA was extracted from peripheral blood using the QIAamp DNA blood midi kit (Qiagen, Hilden, Germany) according to the manufacturer’s protocol. Genomic DNA quality was assessed using Nanodrop (Life technologies, Darmstadt, Germany). The genomic DNA samples were sequenced at Sidra Medicine using an HiSeq 2000 sequencer (Illumina, San Diego, CA, USA). High quality reads were aligned to the human genome reference GRCh37 assembly (hg19). VariantRecalibrator and GATK Haplotypecaller tools were used for variant calling. Sanger sequencing was used to confirm variant by region-specific primers (forward: 5′ TCCAGGCACCCATGTACTTT 3′, reverse: 5′ AGATGGTGATGTAGCGGTCC 3′), which covered the region of interest. Primers were designed using primer3 software (http://primer3.ut.ee/). After confirming the variant identified in WES on first sibling using Sanger sequencing, we targeted sequencing on the second sibling. The second sibling was homozygous for this variant.

### Cell Culture and MC2R and MRAP Vector Expression in HEK293 Cells

The full-length human MC2R wild-type (WT) (NM_001291911.1) and MC2R mutant cDNA cloned in pcDNA3.1-C-(k)DYK vector were purchased from GenScript. Human MRAP clone cDNA clone expression plasmid cloned in pCMV3-MRAP-OFPSpark was purchased from (Sino Biological). HEK293A cells were maintained in a complete Dulbecco’s Modified Eagle Medium (DMEM) with 10% fetal bovine serum (FBS) and 1% penicillin/streptomycin mix in 5% CO_2_ at 37 °C in a humidified incubator. Cells were seeded in a 6-well plate; 24 hours after seeding, cells were transfected using FuGENE HD (Promega) at approximately 80% confluency with total DNA of 3 μg of DNA per well (duplicate) with both wild-type and mutant vectors. Then 48 hours after transfection, the transfected cells were placed into complete DMEM media containing 400 μg/mL of geneticin selective antibiotics (G418 sulfate) (Thermo Fisher Scientific) to select for G418-resistant cells. After 4 weeks, the cells were harvested to assay the expression of the clones to enable us to estimate the relative amount of expression in transfected cells in comparison to the endogenous (untransfected HEK293 cells) expression using real-time polymerase chain rection (PCR).

### Real-Time PCR of MC2R Plasmid Clones

Total RNA was isolated from transfected (wild-type and mutant) and untransfected (endogenous control) HEK293 cells using RNeasy mini kit (Qiagen). The RNA concentration was quantified using Nanodrop. First-strand cDNA was synthesized from 1 μg RNA retrotranscribed using SuperScript III First Strand Synthesis System (Thermo Fisher Scientific). Two μL of the reverse transcribed cDNA was used for real-time PCR using Power SYBR Green Master Mix (Thermo Fisher Scientific). Gene-specific primers (rtMC2R-forward: 5′TCCAGGCACCCATGTACTTT3′; rtMC2R-reverse: 5′GTCATCGGCTGTGGTTTCAA3′ [166 bp] and rtGAPDH forward: 5′CAGCCTCAAGATCATCAGCA3′, rtGAPDH reverse: 5′GGTCATGAGTCCTTCCACGA3′ [103 bp]) were used for PCR amplification. The amplification reactions were carried out in QuantStudio 12K flex real-time PCR system (Applied Biosystems, ABI). Samples were measured in triplicates and mRNA levels were normalized against GAPDH using ΔΔCT method [16].The data presented are means ± SE with *P < 0.05 and **P < 0.01.

### ACTH Stimulation and cAMP Assay

Stably transfected HEK293 cells with the wild-type MC2R-WT and MC2R-mutant were seeded in a 6-well plate and grown until they reached approximately 80% confluent. After 80% confluency both wild-type and mutant cells were transiently co-transfected with 3 μg of MRAP DNA per well. Cells were assayed for cAMP assay experiment after 48 hours of co-transfection with MRAP. Cells were incubated with Opti-MEM reduced-serum medium (Gibco) for 2 hours, in the presence of 3-isobutyl-1-methylxanthine (IBMX) at a concentration of 1 mmol/L. After a starvation period of 2 hours, the cells were stimulated with ACTH-(1–24) (Sigma-Aldrich) with different concentrations per well (10^-12^ to 10^-5^ mol/L) for 3 hours. After the incubation, cells and media were harvested, heated, and were used for cAMP assay according to a previously established method [17]. The cAMP ELISA assay was performed using cAMP Direct Immunoassay Kit (BioVision, Cat# K371, RRID:AB_2909598). Acetylated protocol was applied for the assay following the manufacturer’s guidelines. All the expression results were normalized to protein concentration using BCA protein assay kit (Thermo Fisher Scientific).

### Statistical Analysis

For protein expression and cAMP production measurement assays, results are presented for duplicates as mean ± SD using cells from 2 independent experiments. One-way ANOVA was performed using GraphPad Prism version 8.02 (GraphPad Software, San Diego, California).

## Results

**Figure 1.**
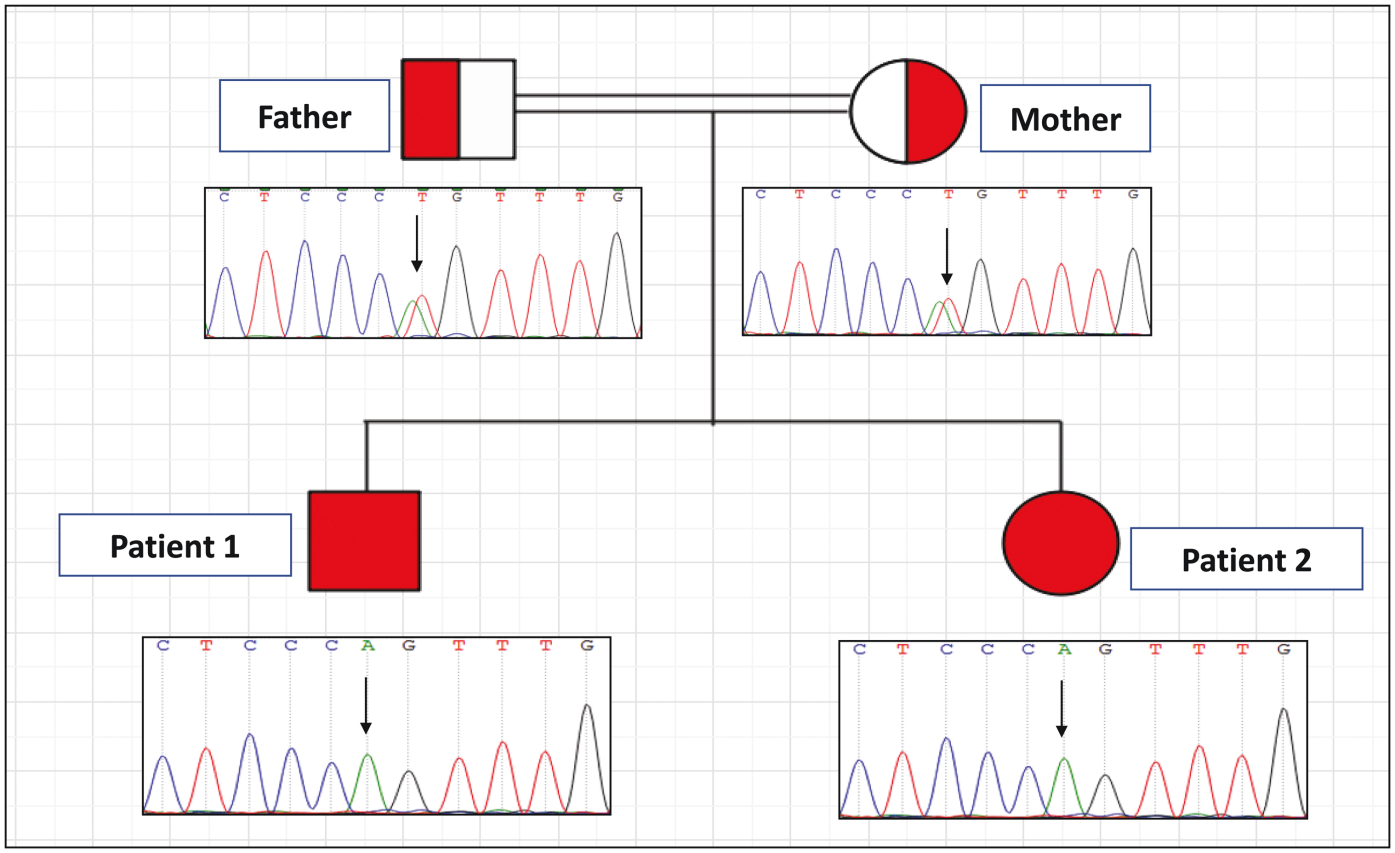
Pedigree diagram showing consanguineous parents with 2 affected children. Sanger sequencing demonstrating the c.326T>A homozygous mutation in both patients (lower panel) and heterozygous genotype in both parents (upper panel). Black arrow indicates the nucleotide position C.326.

Whole exome sequencing of the 2 patients revealed a novel homozygous missense mutation, a substitution of thymine by adenine (c.326T>A), located within exon 2 of the *MC2R* gene, whereas the parents were carriers for this mutation. This single base pair resulted in a substitution of leucine (nonpolar, strong hydrophobic side chain) by glutamine (polar, strong hydrophilic side chain) at position 109 (p.Leu109Gln). This mutation overlaps with the 2 transcripts of MC2R and resides in the third transmembrane domain of the gene. We confirmed the homozygous T to A transversion mutation detected by WES using Sanger sequencing. The parents were heterozygous carriers for this mutation (Fig. 1). Variant effect prediction tools: SIFT (https://sift.bii.a-star.edu.sg/), Polyphen2 (http://genetics.bwh.harvard.edu/pph2/), and MutationTaster (http://www.mutationtaster.org) predicted this mutation to be deleterious, probably damaging, and disease causing, respectively. This variant was considered as a novel mutation because it was not found in the ExAC (http://exac.broadinstitute.org/) or in 1000Genomes phase-3 (http://www.1000genomes.org/) databases.

### Mutant MC2R Cells Generate Decreased cAMP When Stimulated With ACTH

**Figure 2.**
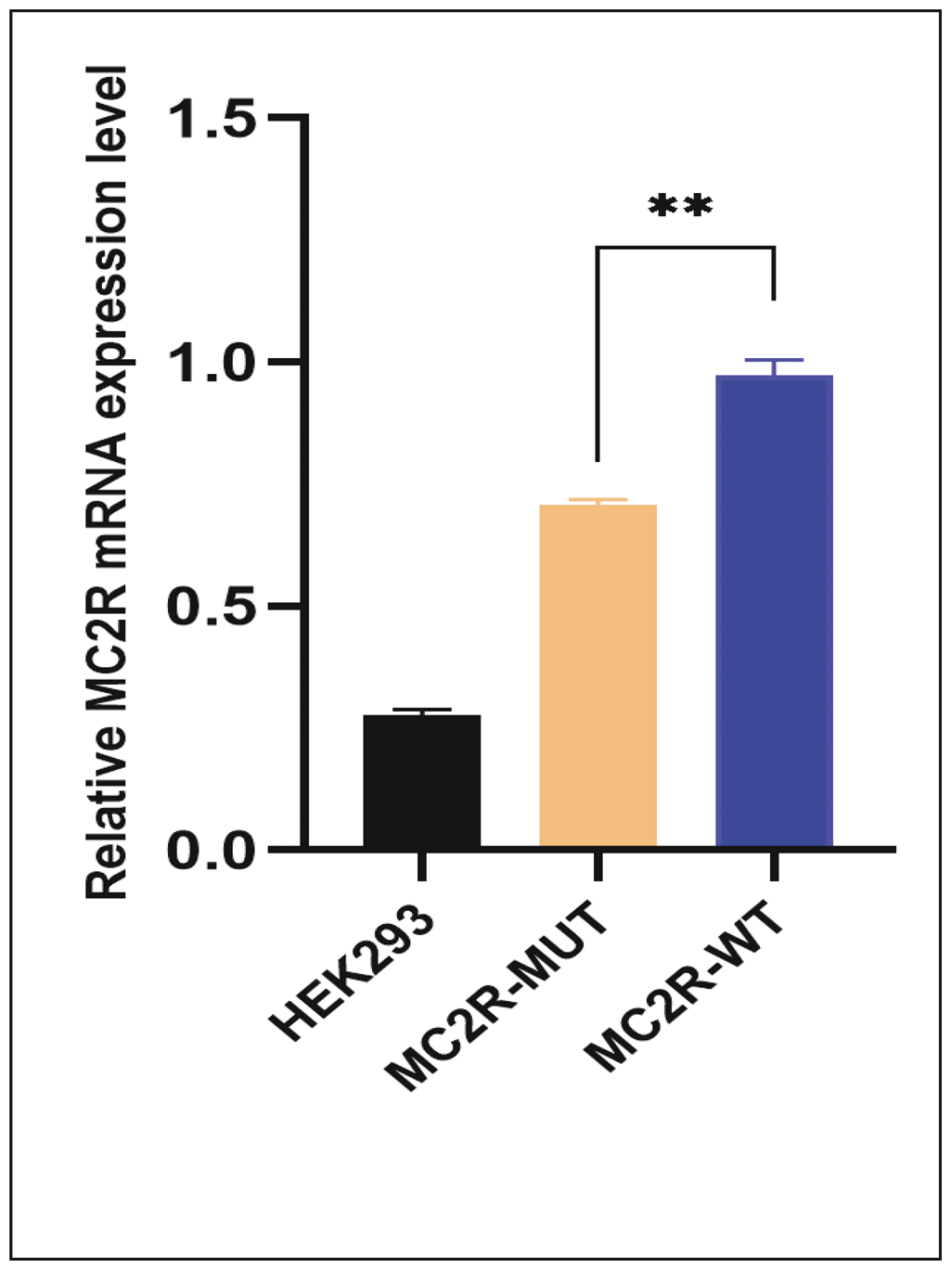
Real-time PCR analysis MC2R mRNA expression levels from HEK293 cells transfected with wild-type or mutant (L109Q) clones, untransfected HEK293 cells were used as basal control, * indicates groups are significantly different (*P* < 0.0001, one-way ANOVA).

**Figure 3.**
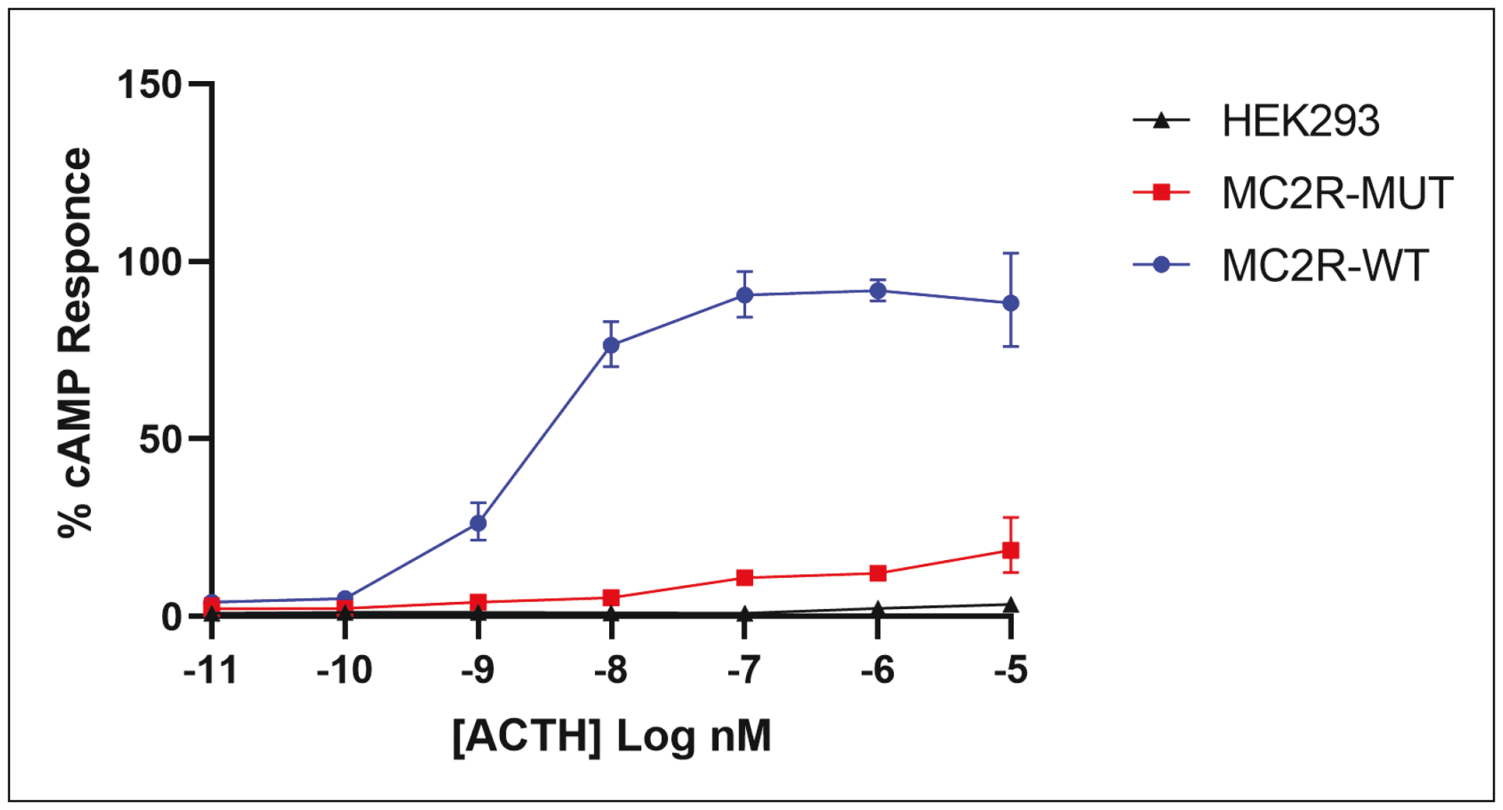
Dose response of ACTH-mediated cAMP production measurement in HEK293 cells, HEK293 cells transfected with either MC2R wild-type or MC2R mutant (L109Q) clone. Untransfected HEK293 cells were used as endogenous negative control for basal cAMP production.

We noticed reduced protein expression of the mutant p.Leu109GLn compared with the wild-type expressing cells. We further assessed the expression using real-time PCR, and the mutant cells showed decreased mRNA expression (Fig. 2). After confirming reduced mRNA and protein in the mutant cells we assessed the cAMP levels in wild-type, mutant (co-transfected with MC2R and MRAP clones), and untransfected HEK293 cells as a negative control. Mutant cells resulted in generation of very low cAMP in comparison with wild-type cells (Fig. 3).

## Discussion

In this study, we report the clinical findings and in vitro functional analysis of a novel MC2R mutation in 2 siblings, born to a healthy consanguineous family. The 2 patients presented with the typical phenotype of FGD-1 with hypoglycemia, recurrent infections, high plasma ACTH, and low serum cortisol level. We identified a homozygous novel mutation, c.326T>A, p.Leu109Glu in the *MC2R* gene using WES. Leucine 109 is located in the third transmembrane domain of the 7 transmembrane domains of the GPCR. This third transmembrane domain is important and plays a critical role in ligand selectivity and potency for receptor activation [18]. Another unique feature of this domain is that it is the least exposed to lipid membrane, buried inside compared with the GPCR domains [19]. Previously reported mutations in Human Gene Mutation Database (HGMD) in the third transmembrane domain of *MC2R* gene showed decreased cAMP production. Variants p.Asp103Asn, p.Asp107Asn, and p.Arg128Cys impair ACTH receptor function and result in significant impairment of cAMP generation; a Thy129Cys mutant caused significant intracellular retention, and p.Ile130Asn exhibited partial trafficking impairment [20]. Tsigos and colleagues showed that the p.Ser120Arg variant leads to ACTH resistance [21]. The variant p.Ala126Ser alters MC2R structure and function [22].

To examine the effect of the novel mutation (p.Leu109Gln), we undertook in vitro functional analysis by transfecting wild-type and mutant MC2R clones in HEK293 cells. We stimulated HEK293 cells with ACTH(1-24) with different concentrations, ranging from ACTH 12 mol/L to 6 mol/L to measure cAMP generation. The analysis of duplicate run samples showed very low cAMP generation when stimulated with ACTH compared to wild-type cell lines, indicating impaired function of the mutant ACTH receptor. This result confirms that the p.Leu109Gln mutation affects signaling pathways due to a defect in generating a secondary messenger cAMP, consequently leading to ACTH resistance. However, under normal physiology, downstream signaling of cAMP plays a crucial role in activation of targets such as protein kinase A and cAMP response element-binding protein (CREB), which regulate transcription of genes involved in steroidogenic enzymes synthesis and secretion [13].

Since the mode of inheritance of the *MC2R* gene is autosomal recessive, the frequency should be higher in societies such as among the Arabs which have a long tradition of consanguinity [23]. However, to our knowledge, there are only 3 studies of *MC2R* gene mutations in the literature from the Arab countries. The first study was by Chan and colleagues [24], which reported a patient of Saudi Arabian origin carrying double homozygous *MC2R* mutations (Y129C and F278C) who was diagnosed with FGD and presented with spastic quadriplegia due to hypoglycemic episodes. The second study identified *MC2R* mutations in 3 patients of Arab origin: Kuwaiti, Lebanese, and Sudanese backgrounds. The patients from Kuwaiti and Sudanese origin carried frameshift mutations, c. 459_460insC (p.I154fsX248), and c.539C>(p.S180X) respectively. The Lebanese origin patient carried a nonsense mutation c.539C>(p.S180X) [25]. In the third study, Kandari et al reported a homozygous point mutation in the *MC2R* gene in 5 Arab kindreds from Saudi Arabia and Kuwait. All the 5 patients carried a common mutation c.459_460insC resulting in a frameshift at amino acid position 248 (p.I154fsX248) [26]. The lack of reported cases in the literature in this consanguineous population could be due to misdiagnosis of FGD.

Since we identified the genetic abnormality in the first sibling using WES, it was easy to make the diagnosis very early on the second sibling and to start treatment early. With delayed diagnosis, inadequate treatment, or if left untreated, FGD leads to fatal complications or severe mental disabilities [27].

We observed elevated serum TSH levels in patient 2 on day 4 of birth; however, since the patient’s serum T4 level was normal, medication was not started. On repeat testing at 2 months of age, the patient had normal serum TSH and T4 levels. The plasma aldosterone level showed an elevated levels only in patient 2 during her neonatal period, and the normal reference range for plasma aldosterone is very wide as well. Therefore, we do not think this finding is associated with the *MC2R* mutation in our patient. Serum insulin as well C-peptide in patient 1 was undetectable. This is in keeping with our finding, since *MC2R* mutations are not associated with hyperinsulinemia hypoglycemia.

Our first patient has hypothyroidism and as far as we know there are only few cases of *MC2R* mutations and hypothyroidism. All the 5 patients reported by Al Kandari et al with homozygous *MC2R* mutation c.459_560insC presented with thyroid dysfunction ranging from neonatal thyroid dysfunction to subclinical hypothyroidism [26]. Another study reported a Caucasian girl with a mutation in *MC2R* p.S74I, diagnosed with primary hypothyroidism [28]. Recently, Heshmatzad et al reported a 2-year-old boy with *MC2R* mutation, who presented with hypothyroidism and congenital adrenal hypoplasia [29]. The link between MC2R and hypothyroidism is not known yet; future investigation is needed to elucidate the underlying mechanism responsible for hypothyroidism.

## Data Availability

Some or all datasets generated during and/or analyzed during the current study are not publicly available but are available from the corresponding author on reasonable request.

## Abbreviations

ACTH: adrenocorticotropic hormone
cAMP: cyclic adenosine monophosphate
DMEM: Dulbecco’s Modified Eagle Medium
FGD: familial glucocorticoid deficiency
GPCR: G protein coupled receptor
MC2R: melanocortin-2 receptor
MRAP: melanocortin-2 receptor accessory protein
PCR: polymerase chain reaction
TSH: thyrotropin (thyroid-stimulating hormone)
WES: whole exome sequencing
